# 6-Chloro-3-(3-methyl­phen­yl)-1,2,4-triazolo[4,3-*b*]pyridazine

**DOI:** 10.1107/S1600536811035288

**Published:** 2011-09-03

**Authors:** Jasmin Preis, Dieter Schollmeyer, Heiner Detert

**Affiliations:** a University Mainz, Duesbergweg 10-14, 55099 Mainz, Germany

## Abstract

The title compound, C_12_H_9_ClN_4_, was prepared from dichloro­pyridazine and tolyl­tetra­zole in a nucleophilic biaryl coupling followed by thermal ring transformation. The mol­ecule is essentially planar (r.m.s. deviation for all non-H atoms = 0.036 Å) and an intra­molecular C—H⋯N hydrogen bond occurs. In the crystal, the mol­ecules form dimers connected *via* π–π inter­actions [centroid–centroid distance = 3.699 (2) Å], which are further connected to neighbouring mol­ecules *via* C—H—N bonds. The bond lengths in the pyridazine ring system indicate a strong localization of the double bonds and there is a weak C—Cl bond [1.732 (3) Å].

## Related literature

The acyl­ation of tetra­zoles with chloro­azines and thermal ring transformation leads to triazolo annulated azines, see: Huisgen *et al.* (1961 ▶); Glang *et al.* (2008 ▶). For two benzo-annulated triazolopyridazines, see: Boulanger *et al.* (1991 ▶). For a highly phenyl­ated triazolopyrazine, see: Kozhevnikov *et al.* (2005 ▶)·For the synthesis of higher conjugated and annulated heterocyclic π-systems see: Detert & Schollmeyer (1999 ▶); Sugiono & Detert (2001 ▶). For the synthesis of 1,3,4-oxadiazo­les and triazoles, see: Huisgen, Sauer & Seidel (1960 ▶); Huisgen, Sturm & Markgraf (1960 ▶) and of triazolo-annulated azines, see: Preis *et al.* (2011 ▶).
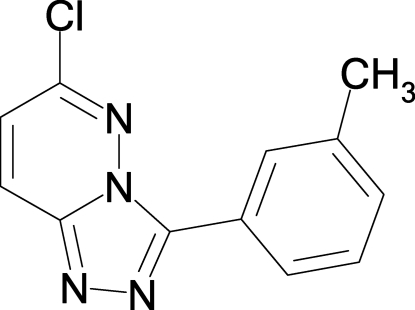


## Experimental

### 

#### Crystal data

C_12_H_9_ClN_4_
*M* *_r_* = 244.68
Monoclinic, 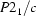

*a* = 7.1001 (18) Å
*b* = 11.431 (3) Å
*c* = 13.783 (3) Å
β = 93.403 (6)°
*V* = 1116.6 (5) Å^3^
*Z* = 4
Mo *K*α radiation
μ = 0.32 mm^−1^
*T* = 173 K
0.60 × 0.05 × 0.05 mm

#### Data collection

Bruker SMART APEXII diffractometer
14031 measured reflections
2664 independent reflections
1226 reflections with *I* > 2σ(*I*)
*R*_int_ = 0.130

#### Refinement

*R*[*F* ^2^ > 2σ(*F* ^2^)] = 0.050
*wR*(*F* ^2^) = 0.132
*S* = 0.84
2664 reflections
155 parameters
H-atom parameters constrained
Δρ_max_ = 0.48 e Å^−3^
Δρ_min_ = −0.26 e Å^−3^

### 

Data collection: *APEX2* (Bruker, 2006 ▶); cell refinement: *SAINT* (Bruker, 2006 ▶); data reduction: *SAINT*; program(s) used to solve structure: *SIR97* (Altomare *et al.*, 1999 ▶); program(s) used to refine structure: *SHELXL97* (Sheldrick, 2008 ▶); molecular graphics: *PLATON* (Spek, 2009 ▶); software used to prepare material for publication: *PLATON*.

## Supplementary Material

Crystal structure: contains datablock(s) I, global. DOI: 10.1107/S1600536811035288/bt5632sup1.cif

Structure factors: contains datablock(s) I. DOI: 10.1107/S1600536811035288/bt5632Isup2.hkl

Supplementary material file. DOI: 10.1107/S1600536811035288/bt5632Isup3.cml

Additional supplementary materials:  crystallographic information; 3D view; checkCIF report

## Figures and Tables

**Table 1:** Hydrogen-bond geometry (Å, °)

*D*—H⋯*A*	*D*—H	H⋯*A*	*D*⋯*A*	*D*—H⋯*A*
C6—H6⋯N9^i^	0.95	2.55	3.344 (4)	141
C11—H11⋯N2	0.95	2.34	3.006 (4)	127

Symmetry code: (i) 
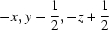
.

**Fig. 1.**
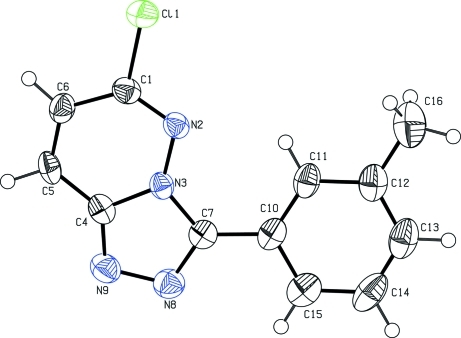
View of compound I. Displacement ellipsoids are drawn at the 50% probability level.

## supplementary crystallographic information

### Comment

The title compound was synthesized as part of a larger project focusing on the synthesis of higher conjugated and annulated heterocyclic π-systems (Detert & Schollmeyer, 1999; Sugiono & Detert, 2001). The acylation of tetrazoles followed by thermal ring transformation is a highly efficient route for the synthesis of 1,3,4-oxadiazoles and triazoles (Huisgen, Sauer & Seidel, 1960; Huisgen, Sturm & Markgraf, 1960) and can also be applied to 2-chloroazines to yield triazolo-annulated azines (Preis *et al.*, 2011). In the crystal the title compound adopts an essentially planar structure with a dihedral angle of 2.21° between the mean planes of the phenyl ring and the bicyclic system and deviations of less than 0.01 Å from the least square plane. All torsion angles in the C—N-framework are below 2°; the torsion angle of -176.5 (3)° (C16—C12—C13—C14) results from methyl substitution. With 1.372 (3)Å (N2—N3) and 1.381 (3)Å (N8 - N9), the N—N bonds in the bicyclic framework are significantly longer than the C—N bonds C1—N2 (1.290 (4) Å), C4 - N9 (1.317 (4) Å), and C7 - N8 (1.324 (4) Å). This, the longer bonds N3—C4 (1.383 (4) Å) and N3 - C7 (1.378 (4) Å) and the alternating C—C bond lengths in the pyridazine (C4 - C5: 1.416 (4) Å; C5 - C6: 1.3435 (4) Å; C1 - C6: 1.426 (4) Å) indicate a strong localization of the double bonds. Contrary to the short bond C1 - N2 (1.290 (4) Å), the C1 - Cl1 bond (1.732 (3) Å) is long. This correlates with the reactivity of the C1—Cl1 bond, similar to an imidoyl chloride. Two molecules are connected *via* a center of inversion (symmetry operator: 1-x, 1-y, 1-z), by π-π-interactions and hydrogen bridging stabilize the lattice. The distances of the centroids of pyridazine and tolyl rings are only 3.70 Å and C—H—N bonds between C6—H6—N9 (H6—N9: 2.55 Å) connect the molecules.

### Experimental

The title compound was prepared by adding pyridine (0.89 g, 10 mmol) to a solution of 3,6-dichloropyridazine (0.45 g, 3 mmol) and 5-(3-methyl- phenyl)tetrazole (0.96 g, 9 mmol) in toluene (15 ml). The mixture was heated to relflux for 5 h, cooled, filtered, and concentrated. The residue was purified by chromatography (SiO_2_ /toluene/ethyl acetate = 1/1, *R*_f_ = 0,23). The title compound was isolated as an off-white powder with m.p. = 422 - 425 K. Crystals were obtained by slow evaporation of a solution of the title compound in chloroform/hexanes.

### Refinement

Hydrogen atoms attached to carbons were placed at calculated positions with C—H = 0.95 Å (aromatic) or 0.98–0.99 Å (*sp*^3^ C-atom). All H atoms were refined in the riding-model approximation with isotropic displacement parameters  set at 1.2–1.5 times of the *U*_eq_ of the parent atom.

### Crystal data

**Table:**

C_12_H_9_ClN_4_	*F*(000) = 504
*M**_r_* = 244.68	*D*_x_ = 1.456 Mg m^−^^3^
Monoclinic, *P*2_1_/*c*	Mo *K*α radiation, λ = 0.71069 Å
Hall symbol: -P 2ybc	Cell parameters from 1195 reflections
*a* = 7.1001 (18) Å	θ = 2.3–20.2°
*b* = 11.431 (3) Å	µ = 0.32 mm^−^^1^
*c* = 13.783 (3) Å	*T* = 173 K
β = 93.403 (6)°	Needle, colourless
*V* = 1116.6 (5) Å^3^	0.60 × 0.05 × 0.05 mm
*Z* = 4	

### Data collection

**Table:**

Bruker SMART APEXII diffractometer	1226 reflections with *I* > 2σ(*I*)
Radiation source: sealed Tube	*R*_int_ = 0.130
graphite	θ_max_ = 27.9°, θ_min_ = 2.3°
CCD scan	*h* = −9→9
14031 measured reflections	*k* = −15→14
2664 independent reflections	*l* = −18→18

### Refinement

**Table:**

Refinement on *F*^2^	Primary atom site location: structure-invariant direct methods
Least-squares matrix: full	Secondary atom site location: difference Fourier map
*R*[*F*^2^ > 2σ(*F*^2^)] = 0.050	Hydrogen site location: inferred from neighbouring sites
*wR*(*F*^2^) = 0.132	H-atom parameters constrained
*S* = 0.84	*w* = 1/[σ^2^(*F*_o_^2^) + (0.056*P*)^2^] where *P* = (*F*_o_^2^ + 2*F*_c_^2^)/3
2664 reflections	(Δ/σ)_max_ < 0.001
155 parameters	Δρ_max_ = 0.48 e Å^−^^3^
0 restraints	Δρ_min_ = −0.26 e Å^−^^3^

### Special details

**Table:**

Experimental. ^1^H-NMR (300 MHz,CDCl_3_): 8.23 (m, 2 H, 2-H, 6-H, ph), 8.16 (d, 1 H, J = 9.6 Hz, 5-H pyr), 7.41 (t, 1 H, 5-H, ph), 7.32 (d, J = 8.2 Hz, 1 H. 4-H, ph), 7.13 (d, 1 H, J = 9.6 Hz, 4-H pyr), 2.52 (s, 3 H, CH_3_). ^13^C-NMR (75 MHz,CDCl_3_): 149.1 (Cq), 148.2 (Cq), 143.5 (Cq), 139.0 (Cq), 136.6 (Cq), 131.6 (CH), 128.7 (CH), 128.3 (CH), 126.6 (CH), 124.7 (CH), 122.0 (CH), 21.5 (CH_3_). FD-MS: 244.3 (M^++^, 100%, Cl-pattern).
Geometry. All e.s.d.'s (except the e.s.d. in the dihedral angle between two l.s. planes) are estimated using the full covariance matrix. The cell e.s.d.'s are taken into account individually in the estimation of e.s.d.'s in distances, angles and torsion angles; correlations between e.s.d.'s in cell parameters are only used when they are defined by crystal symmetry. An approximate (isotropic) treatment of cell e.s.d.'s is used for estimating e.s.d.'s involving l.s. planes.
Refinement. Refinement of *F*^2^ against ALL reflections. The weighted *R*-factor *wR* and goodness of fit *S* are based on *F*^2^, conventional *R*-factors *R* are based on *F*, with *F* set to zero for negative *F*^2^. The threshold expression of *F*^2^ > σ(*F*^2^) is used only for calculating *R*-factors(gt) *etc*. and is not relevant to the choice of reflections for refinement. *R*-factors based on *F*^2^ are statistically about twice as large as those based on *F*, and *R*- factors based on ALL data will be even larger.

### Fractional atomic coordinates and isotropic or equivalent isotropic displacement parameters (Å^2^)

**Table:**

	*x*	*y*	*z*	*U*_iso_*/*U*_eq_	
Cl1	0.14497 (13)	0.12426 (7)	0.55906 (6)	0.0543 (3)	
C1	0.1347 (4)	0.2284 (3)	0.4676 (2)	0.0351 (7)	
N2	0.1881 (3)	0.3314 (2)	0.49595 (17)	0.0319 (6)	
N3	0.1769 (3)	0.41168 (19)	0.42183 (16)	0.0300 (6)	
C4	0.1166 (4)	0.3900 (3)	0.3262 (2)	0.0346 (7)	
C5	0.0608 (4)	0.2744 (3)	0.3013 (2)	0.0374 (7)	
H5	0.0189	0.2548	0.2366	0.045*	
C6	0.0691 (4)	0.1934 (3)	0.3722 (2)	0.0373 (7)	
H6	0.0321	0.1148	0.3590	0.045*	
C7	0.2199 (4)	0.5292 (2)	0.4268 (2)	0.0349 (7)	
N8	0.1856 (4)	0.5739 (2)	0.3389 (2)	0.0432 (7)	
N9	0.1210 (4)	0.4874 (2)	0.27542 (18)	0.0432 (7)	
C10	0.2901 (4)	0.5958 (2)	0.5118 (2)	0.0377 (7)	
C11	0.3162 (4)	0.5480 (3)	0.6046 (2)	0.0416 (8)	
H11	0.2890	0.4676	0.6143	0.050*	
C12	0.3820 (4)	0.6163 (3)	0.6838 (3)	0.0462 (8)	
C13	0.4225 (4)	0.7318 (3)	0.6691 (3)	0.0534 (10)	
H13	0.4694	0.7782	0.7224	0.064*	
C14	0.3961 (5)	0.7824 (3)	0.5778 (3)	0.0576 (11)	
H14	0.4227	0.8630	0.5690	0.069*	
C15	0.3300 (4)	0.7138 (3)	0.4989 (3)	0.0493 (9)	
H15	0.3123	0.7479	0.4362	0.059*	
C16	0.4006 (6)	0.5656 (3)	0.7827 (3)	0.0736 (12)	
H16A	0.4618	0.4889	0.7802	0.110*	
H16B	0.2752	0.5566	0.8079	0.110*	
H16C	0.4771	0.6176	0.8256	0.110*	

### Atomic displacement parameters (Å^2^)

**Table:**

	*U*^11^	*U*^22^	*U*^33^	*U*^12^	*U*^13^	*U*^23^
Cl1	0.0708 (6)	0.0485 (5)	0.0425 (5)	−0.0143 (4)	−0.0053 (4)	0.0108 (4)
C1	0.0308 (16)	0.0411 (18)	0.0331 (19)	−0.0015 (13)	−0.0003 (14)	0.0048 (13)
N2	0.0285 (13)	0.0389 (14)	0.0279 (14)	−0.0026 (11)	−0.0009 (11)	0.0018 (10)
N3	0.0262 (13)	0.0361 (14)	0.0274 (14)	−0.0001 (10)	−0.0003 (10)	−0.0025 (10)
C4	0.0278 (15)	0.0449 (18)	0.0308 (17)	0.0044 (13)	0.0007 (13)	−0.0017 (14)
C5	0.0328 (17)	0.0533 (19)	0.0254 (17)	−0.0003 (14)	−0.0048 (14)	−0.0088 (14)
C6	0.0326 (17)	0.0400 (18)	0.039 (2)	−0.0066 (13)	−0.0005 (14)	−0.0060 (14)
C7	0.0285 (16)	0.0368 (17)	0.0395 (19)	0.0028 (13)	0.0039 (14)	−0.0014 (13)
N8	0.0455 (16)	0.0393 (15)	0.0446 (17)	0.0024 (12)	0.0010 (13)	0.0038 (13)
N9	0.0479 (16)	0.0456 (16)	0.0356 (16)	0.0028 (13)	−0.0004 (13)	0.0048 (12)
C10	0.0263 (16)	0.0366 (18)	0.051 (2)	−0.0002 (13)	0.0081 (14)	−0.0097 (14)
C11	0.0310 (17)	0.0449 (19)	0.049 (2)	−0.0027 (14)	0.0000 (15)	−0.0131 (15)
C12	0.0322 (17)	0.056 (2)	0.051 (2)	−0.0002 (16)	0.0019 (15)	−0.0142 (17)
C13	0.0346 (19)	0.060 (2)	0.066 (3)	−0.0061 (16)	0.0072 (18)	−0.024 (2)
C14	0.039 (2)	0.044 (2)	0.090 (3)	−0.0096 (16)	0.013 (2)	−0.018 (2)
C15	0.0389 (19)	0.045 (2)	0.064 (3)	0.0013 (15)	0.0091 (18)	−0.0033 (17)
C16	0.073 (3)	0.080 (3)	0.065 (3)	0.004 (2)	−0.012 (2)	−0.018 (2)

### Geometric parameters (Å, °)

**Table:**

Cl1—C1	1.732 (3)	C10—C15	1.392 (4)
C1—N2	1.290 (4)	C10—C11	1.393 (4)
C1—C6	1.426 (4)	C11—C12	1.399 (4)
N2—N3	1.372 (3)	C11—H11	0.9500
N3—C7	1.378 (4)	C12—C13	1.369 (5)
N3—C4	1.383 (4)	C12—C16	1.481 (5)
C4—N9	1.317 (4)	C13—C14	1.387 (5)
C4—C5	1.416 (4)	C13—H13	0.9500
C5—C6	1.345 (4)	C14—C15	1.399 (5)
C5—H5	0.9500	C14—H14	0.9500
C6—H6	0.9500	C15—H15	0.9500
C7—N8	1.324 (4)	C16—H16A	0.9800
C7—C10	1.460 (4)	C16—H16B	0.9800
N8—N9	1.381 (3)	C16—H16C	0.9800
			
N2—C1—C6	127.5 (3)	C11—C10—C7	123.5 (3)
N2—C1—Cl1	114.0 (2)	C10—C11—C12	121.1 (3)
C6—C1—Cl1	118.4 (2)	C10—C11—H11	119.4
C1—N2—N3	112.4 (2)	C12—C11—H11	119.4
N2—N3—C7	127.7 (2)	C13—C12—C11	119.1 (3)
N2—N3—C4	126.2 (2)	C13—C12—C16	120.4 (3)
C7—N3—C4	106.1 (2)	C11—C12—C16	120.5 (3)
N9—C4—N3	109.8 (2)	C12—C13—C14	121.1 (3)
N9—C4—C5	132.4 (3)	C12—C13—H13	119.4
N3—C4—C5	117.7 (3)	C14—C13—H13	119.4
C6—C5—C4	117.8 (3)	C13—C14—C15	119.5 (3)
C6—C5—H5	121.1	C13—C14—H14	120.2
C4—C5—H5	121.1	C15—C14—H14	120.2
C5—C6—C1	118.3 (3)	C10—C15—C14	120.3 (4)
C5—C6—H6	120.8	C10—C15—H15	119.8
C1—C6—H6	120.8	C14—C15—H15	119.8
N8—C7—N3	107.6 (3)	C12—C16—H16A	109.5
N8—C7—C10	124.6 (3)	C12—C16—H16B	109.5
N3—C7—C10	127.8 (3)	H16A—C16—H16B	109.5
C7—N8—N9	109.8 (2)	C12—C16—H16C	109.5
C4—N9—N8	106.6 (2)	H16A—C16—H16C	109.5
C15—C10—C11	118.7 (3)	H16B—C16—H16C	109.5
C15—C10—C7	117.7 (3)		
			
C6—C1—N2—N3	−0.1 (4)	C10—C7—N8—N9	179.9 (3)
Cl1—C1—N2—N3	−179.59 (18)	N3—C4—N9—N8	0.1 (3)
C1—N2—N3—C7	179.0 (3)	C5—C4—N9—N8	178.9 (3)
C1—N2—N3—C4	−0.1 (4)	C7—N8—N9—C4	0.1 (3)
N2—N3—C4—N9	178.9 (2)	N8—C7—C10—C15	−2.3 (4)
C7—N3—C4—N9	−0.3 (3)	N3—C7—C10—C15	177.9 (3)
N2—N3—C4—C5	−0.0 (4)	N8—C7—C10—C11	176.7 (3)
C7—N3—C4—C5	−179.3 (2)	N3—C7—C10—C11	−3.1 (5)
N9—C4—C5—C6	−178.4 (3)	C15—C10—C11—C12	−0.2 (4)
N3—C4—C5—C6	0.3 (4)	C7—C10—C11—C12	−179.2 (3)
C4—C5—C6—C1	−0.5 (4)	C10—C11—C12—C13	−0.5 (4)
N2—C1—C6—C5	0.4 (5)	C10—C11—C12—C16	177.2 (3)
Cl1—C1—C6—C5	179.9 (2)	C11—C12—C13—C14	1.2 (5)
N2—N3—C7—N8	−178.9 (2)	C16—C12—C13—C14	−176.5 (3)
C4—N3—C7—N8	0.3 (3)	C12—C13—C14—C15	−1.1 (5)
N2—N3—C7—C10	1.0 (4)	C11—C10—C15—C14	0.4 (4)
C4—N3—C7—C10	−179.9 (3)	C7—C10—C15—C14	179.4 (3)
N3—C7—N8—N9	−0.3 (3)	C13—C14—C15—C10	0.3 (5)

### Hydrogen-bond geometry (Å, °)

**Table:**

*D*—H···*A*	*D*—H	H···*A*	*D*···*A*	*D*—H···*A*
C6—H6···N9^i^	0.95	2.55	3.344 (4)	141
C11—H11···N2	0.95	2.34	3.006 (4)	127
C15—H15···N8	0.95	2.53	2.864 (5)	101

Symmetry codes: (i) −*x*, *y*−1/2, −*z*+1/2.
